# Predicate Structures, Gesture, and Simultaneity in the Representation of Action in British Sign Language: Evidence From Deaf Children and Adults

**DOI:** 10.1093/deafed/ent020

**Published:** 2013-05-12

**Authors:** Kearsy Cormier, Sandra Smith, Zed Sevcikova

**Affiliations:** ^1^Deafness, Cognition and Language Research Centre, University College London; ^2^Centre for Deaf Studies, University of Bristol

## Abstract

British Sign Language (BSL) signers use a variety of structures, such as constructed action (CA), depicting constructions (DCs), or lexical verbs, to represent action and other verbal meanings. This study examines the use of these verbal predicate structures and their gestural counterparts, both separately and simultaneously, in narratives by deaf children with various levels of exposure to BSL (ages 5;1 to 7;5) and deaf adult native BSL signers. Results reveal that all groups used the same types of predicative structures, including children with minimal BSL exposure. However, adults used CA, DCs, and/or lexical signs simultaneously more frequently than children. These results suggest that simultaneous use of CA with lexical and depicting predicates is more complex than the use of these predicate structures alone and thus may take deaf children more time to master.

Sign languages such as British Sign Language (BSL) use a variety of structures to represent action and other verbal meanings. One structure is known as constructed action (CA, or enactment, also known as role-shift) where the signer uses his or her body (the head, face, arms, and torso) to represent the thoughts, feelings, or actions of a referent using the surrounding space on a real-world scale. These structures may be highly iconic where the body represents the body, or the facial expression represents the facial expression of a referent, or the hands and arms represent the hands and arms of the referent. Photo 1a shows an example of CA where the signer’s body, arms, hands, and facial expression iconically represent the body of a bear. Photo 1b shows an example where the signer’s hands/arms iconically represent the hand/arm(s) of a referent moving a flat object rightward. (See Appendix for all photos.) CA seems to be a very frequent representational strategy in many sign languages, particularly in narrative but also other genres (cf. de Beuzeville, Johnston, & Schembri, 2009; Quinto-Pozos, 2007a; Quinto-Pozos & Mehta, 2010).

In another structure which we refer to as depicting constructions (DCs, also known as classifier constructions, depicting signs, depicting verbs, or verbs of location and motion), the hand represents the location and/or motion of an entity. Different types of DCs have been described in the literature but we only focus on two particular types here—that is, entity constructions and size and shape specifiers (SASS). Photo 2 shows an example of an entity DC where the signer’s hands represent an upright entity located in a particular position in space. With entity DCs, the hand iconically represents the location and/or motion of a referent (e.g., a person, animal, or vehicle) on a small scale in the signing space in front of the signer. With SASS DCs, the hand(s) depict the size and/or shape of an object by tracing an outline of the object.

Finally, sign languages also use lexical units for expressing verbal meaning. Such lexical verbs do not represent the location or motion of a referent in real space in contrast with DCs. Predicates in BSL and other sign languages may contain one or more of these elements that can be articulated simultaneously or sequentially. The simultaneous use of CA and DCs in particular is fairly well documented (e.g., Aarons & Morgan, 2003; Dudis, 2004; Metzger, 1995; Perniss, 2007; Quinto-Pozos, 2007b; Sallandre, 2007; Sallandre & Cuxac, 2002; Slobin et al., 2003). Much of this previous research has focused on how these simultaneous constructions make systematic use of multiple articulators and/or combine multiple roles/perspectives, which in turn require higher cognitive demand and linguistic skill. Equally, lexical signs can be used simultaneously with CA (typically, lexical verbs produced by the hand(s) and CA produced non-manually) or DCs (e.g., a lexical predicate on one hand and DC on the other). Most previous research in this area has either examined the simultaneous use of CA and DC or has considered CA in the context of quotation or reported action, usually in combination with lexical signs. However, very few studies have sought to distinguish the use of CA with and without simultaneous lexical signs.

This paper examines differences in the frequency and use of these various predicate structures between groups of deaf children with various extent of sign language exposure and deaf adult native signers.

## Background

### Acquisition of Visible Representations of Action and Verbal Meaning

When considering acquisition of verbal structures in deaf children, it is important to remember that language transmission patterns of sign languages are quite different from spoken languages. Only a small minority of deaf children are born to deaf parents and acquire sign language natively (e.g., Mitchell & Karchmer, 2004, report that only around 5% of American deaf children are born to deaf, signing families). Studies on deaf adult signers have shown age-of-acquisition effects at various levels of sign language grammar, including phonology, morphology, syntax, and the lexicon (e.g., Emmorey, Bellugi, Friederici, & Horn, 1995; Newport, 1990).

Most research on sign language acquisition has focused on native signing children. These studies have found that deaf children acquiring sign language from birth from their deaf parents begin using lexical signs, including verbs, around 12 months of age (e.g., Anderson & Reilly, 2002; Newport, 1990). Deaf (and hearing) children produce communicative gestures as early as 8 months of age (Acredolo & Goodwyn, 1988; Fenson et al., 1994; Petitto, 1988, 1992; Volterra & Caselli, 1985). With respect to lexical composition of early sign vocabularies, Anderson and Reilly (2002) showed that, like English-speaking children, nominals dominate the early ASL vocabulary; predicates occupy a much smaller proportion to start with but from about age 2 begin to escalate slowly and steadily.^1^ The deaf children in Anderson and Reilly’s study appeared to have a greater proportion of predicates in their early lexicon than English-speaking children, although they acknowledge that this could be because many of the predicates produced early on by the deaf children were very iconic and looked similar to gestures produced by hearing children (e.g., SLEEP, CLAP).

Previous research on acquisition of CA and whole entity DCs in deaf children shows that development of these structures begins at about 2–3 years of age but progresses slowly (Loew, 1984; Schick, 1987; Supalla, 1982). Slobin et al. (2003) report that the earliest uses of DCs, including handling constructions, are used before age 3 and are heavily gestural with overt use of facial expressions and body movements. Earlier studies by Ellenberger and Steyaert (1978) reported that from age 4;6 children’s depicting verbs become less pantomime-like and more segmentable. However, as de Beuzeville (2006) notes, it is not clear what Ellenberger and Steyaert meant by “pantomime-like” or “segmentable” as these terms were not defined. Other studies have shown that, even by age 8, DCs are not fully mastered (de Beuzeville, 2006; Schick, 1987, 1990) or produced in adult-like fashion (Slobin et al., 2003).^2^ Thus, it seems that the mastery of DCs stands out from the developmental timetable as being much slower than lexical signs and occurring after the age of 8 or 9. Slobin et al. (2003) suggest that after an early phase of fairly successful mastery there is a prolonged phase of learning to use these constructions as a flexible discourse tool. Even by age 12, Slobin et al. (2003) claim, children still struggle with various discourse and pragmatic functions of CA and DCs—separately and also simultaneously. Slobin et al. (2003) argue that children initially use a gestural system to bootstrap their learning of the more conventional sign system and then move seamlessly from one to the other. Given that DCs share some features with gesture used by non-signers (Schembri, Jones, & Burnham, 2005), it remains debatable whether at any given time children are using gestures which happen to resemble the conventional sign system, or whether they are using the conventional sign system which happens to resemble gesture (de Beuzeville, 2006). Indeed, hearing children also use elements of bodily enactment in their gestures and also use gestures in which the hand represents a referent as early as 2 years of age (McNeill, 1992).

Elements of enactment can be found in children’s early signing. Slobin et al. (2003) claim that manipulative and depictive handle handshapes appear to be available to deaf children at a very early point in development (and are also found to be incorporated into gestured actions produced by mothers). Because handling forms enact the action of handling objects, they reach high levels of accuracy with handling forms fairly early on (Schick, 1990). Schick, in her study, reports that DCs with handling handshapes encoding locative transfer of a direct object in space occurred earlier than whole entity DCs or constructions depicting size and shape characteristics (often referred to as SASS).

For the most part, lexical signs, DCs, and CA in deaf children have been studied separately from each other (e.g., Anderson & Reilly, 2002; Loew, 1984; Schick, 1987; Supalla, 1982). One exception is Slobin et al. (2003) who considered acquisition of DCs in various contexts, including periods of CA. Also, Emmorey and Reilly (1998), Reilly (2000), and Reilly, McIntire and Anderson (1994) considered the timing and expression of reference in deaf children’s use of CA with lexical ASL signs. Reilly (2000) and Reilly, McIntire and Anderson (1994) found that it was not until age 6 that deaf children began to use affective expressions consistently and in time with the manual lexical signs (representing utterances) when quoting characters. Emmorey and Reilly (1998) found that deaf children at ages 3, 5, and 7 preferred to use predicates with CA less than adults, with 7-year-olds producing the least number of such predicates.^3^ Emmorey and Reilly attribute the preference for “straight” narration in the 7-year-olds to the development of children’s narratives at that age that results in “structurally more complex but affectively more bland narratives than younger children” (p. 86), which has also been found for hearing children acquiring spoken languages (Berman, 1988; Reilly, 1992). Thus, Emmorey and Reilly also note that deaf children around age 7 still have difficulties combining “dual” perspectives, that is, when the signer as a narrator uses verbs to describe action and the facial expression represents not the affect of the signer but the character whose action is described.

These previous studies provide useful evidence of the developmental path that children take when acquiring these structures. Still, little is known about how children acquire and use these predicate structures, sequentially or simultaneously. Before we consider these three types of predicate structures, it is important to clarify our assumptions about the status of lexical signs, DCs, and CA as lexical, partly lexical, and non-lexical elements (respectively) within the sign language lexicon.

### The Sign Language Lexicon

**Figure 1:**
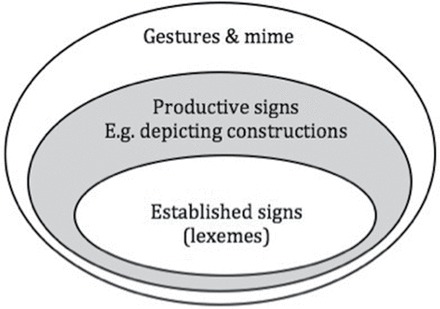
A simplified model of gestural hierarchy and sign typology proposed by Johnston and Schembri (1999).

Johnston and Schembri (1999) proposed a model of the sign lexicon to account for different types of signs occurring in Australian Sign Language (Auslan); a simplified version is shown in Figure 1. The central component of the lexicon, according to Johnston and Schembri, consists of lexemes, also referred to in the literature as the core lexicon (Brentari & Padden, 2001). This core component includes frequently used lexical signs that have been highly standardized and as a result vary minimally in form and meaning. Lexemes contrast with partly lexicalized signs—that is, productive signs. These are signs that are less standardized and conventionalized and can vary to a greater or lesser extent in form and meaning. These include DCs such as whole entity constructions.^4^ Brentari and Padden (2001) refer to these signs as the “non-core” lexicon.

It is clear that productive signs may move into the core lexicon. In such signs, the handshape, location, and/or movement may no longer have a distinct meaning (Aronoff, Meir, Padden, & Sandler, 2003; Supalla, 1986). For example, the lexeme FALL in ASL can be used to refer to objects falling that do not have legs, and it can refer to falls that do not happen directly downward. Johnston and Schembri (1999) note that alternations between lexicalized and decomposed (“de-lexicalized”) forms exist throughout the sign language lexicon. Therefore, a clear distinction between lexical signs and more productive structures can be problematic (Bos, 1990; Engberg-Pedersen, 1993; Johnston, 1991; van Hoek, 1992, 1996).

**Photo 1:**
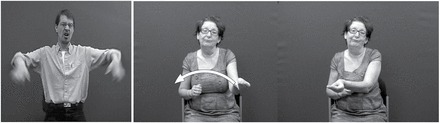
(a) Constructed action. The signer’s body represents the body of a bear (Woll et al., 2004). (b) Handling construction. The signer’s hand represents the hand of a referent moving a flat object rightward (photo originally published in Cormier et al., 2012).

In addition to lexical and partly lexical strategies, signers use non-lexical means, via CA, to portray actions of referents by full or partial mapping of articulators onto actual (or perceived) actions, thoughts, utterances, or feelings. CA can have strong elements of enactment, particularly when it occurs on its own without any co-occurring lexical or partly lexical material (as shown in Photo 1a), which is why CA is represented in Figure 1 as gestures and mime. Just as with partly lexicalized signs, some enacting constructions can become lexicalized in sign languages. This includes lexicalization of handling constructions that involve partial mapping of the articulators, usually arms and hands, onto the arms and hands of the depicted referent, for example, the BSL sign NEWSPAPER, as shown in Photo 3. An example of lexicalized CA without a handling element would be BSL RUN where the arms of the signer represent the arms of someone running.

**Photo 2:**
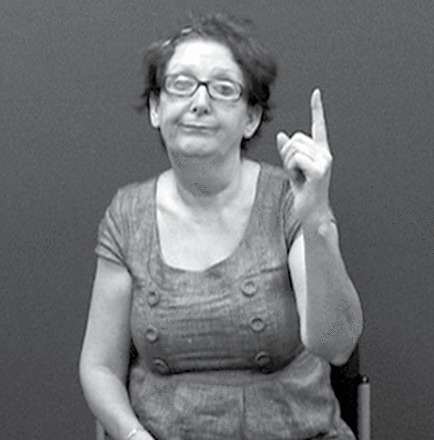
Whole entity construction. The signer’s hand represents an upright entity.

**Photo 3:**
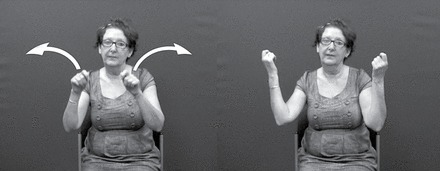
BSL NEWSPAPER (photo originally published in Cormier et al., 2012).

Although it is fairly clear that a sign like BSL RUN can only have become lexicalized from non-lexical CA, the lexical versus non-lexical origins of the lexicalized signs representing handling (e.g., BSL NEWSPAPER as in Photo 3) are less clear. Handling constructions, such as the construction shown in Photo 1b, have been traditionally described in the sign linguistics literature as handling classifier constructions (Schick, 1987; Supalla, 1982; Zwitserlood, 2003, 2012) and placed within the productive, partly lexicalized “non-core” lexicon, similarly to entity constructions (as in Photo 2). However, despite emerging evidence that handling handshapes are discrete and somewhat conventionalized in gestural communication (Sevcikova, 2010), it is unclear whether they are phonemic/morphemic in BSL to the same degree that entity handshapes appear to be (cf., e.g., Schembri et al., 2005). Cormier et al. (2012) have argued that individual tokens that represent handling may fall anywhere along a continuum with non-lexicalized mimetic character viewpoint gesture on one end and fully lexicalized handling signs (such as BSL NEWSPAPER as in Photo 3) on the other. In many cases, it can be very difficult to determine where on the continuum an individual token falls. Therefore, Cormier et al. (2012) propose that it may be more appropriate to err on the side of caution by considering all tokens of depiction of handling (such as Photo 1b) as constructed action/enactment, particularly where the simultaneous use of other CA articulators, such as torso, head, or face, is also involved, unless there is clear evidence of lexicalization to the degree that is found in signs like BSL NEWSPAPER.

The gestural levels of the lexicon described by Johnston and Schembri (1999) include productions that are less constrained by the language and less conventionalized. These productions share similar properties with iconic gestures used by non-signers—in particular, character viewpoint gestures in which the body is used to enact a referent (McNeill, 1992; Parrill, 2010). The lexical level, on the other hand, contains constructions that are more conventionalized and constrained by the linguistic system. Constructions that are only partly lexicalized—for example, in which only one parameter is highly conventionalized—fall somewhere in between. However, even partly lexicalized constructions can look similar in signers and non-signers. Parrill (2010), following McNeill (1992), refers to this type of gesture where the hand represents a referent as an observer viewpoint gesture. Although the handshape used within observer viewpoint gestures may not be as conventionalized as the handshapes that signers use in DCs (Schembri et al., 2005), it is clear that non-signers do use observer viewpoint gestures, where the hand represents a referent, and also character viewpoint (enacting) gestures, which may include the use of any/all bodily articulators. Furthermore, as noted above, both types of viewpoint gesture are used by hearing children as early as 2 years of age (McNeill, 1992).

Representation of action in sign languages can occur at any of the three levels noted above, for example, lexical verb signs, DCs (with more or less conventionalized elements), or CA. A predicate may consist of lexical sign(s) alone (e.g., a verb phrase consisting of lexical verb, adjective, or noun signs), a DC alone (DCs are also known as classifier constructions or classifier predicates), and/or a token of CA alone. More commonly in actual discourse, predicates consist of a combination of tokens of one or more of these different types (whether sequential or simultaneous).

### Predicates and Clauses in Sign Languages

Here we assume Van Valin and La Polla’s (1997) notion of a clause—that is, a language-specific grammatical construction with a universal semantic structure that consists of a predicative element, argument(s) of predicate, and adjunct modifiers of predicate and argument. Specifically, a clause contains a nucleus that should be some kind of a predicating element. Spoken language predicates most commonly include verbs (Van Valin & LaPolla, 1997) and/or other predicative elements, such as predicate nominative or predicate adjective. In sign languages, forms marking argument properties can be lexical verbs (i.e., lexemes that function as verbs; see Figure 1) but also other partially lexicalized or non-lexical verbal structures, for example, DCs or CA (i.e., “productive signs” and “gesture and mime” layers in Figure 1). A predicate thus includes a verbal or non-verbal element and any complements (which may include embedded clauses).

In sign language discourse, a clause may consist only of a single predicate. Each predicative element can thus represent a separate clause, even if there are no explicit and separate elements for the various arguments of the verb (Johnston, Vermeerbergen, Schembri, & Leeson, 2007).

In summary, in sign languages, a range of structures can be predicative in nature, ranging from *lexical items*, which are highly conventionalized in form and meaning, to *whole entity constructions*, which have some conventional components but some non-conventional (productive) components, to *constructed action* as being very unconventionalized in form and meaning. Furthermore, these elements can be combined together, simultaneously or sequentially. However, it is unclear how signers with varying levels of BSL skill use and combine such elements together to express action and verbal meaning. It is also not clear how much sign language exposure is required for these various structures to emerge. Although the most conventionalized structures (i.e., lexical items) are conventionalized in that they are shared by a language community, partly lexicalized structures (i.e., DCs) and non-lexicalized structures (i.e., CA) share so much with gesture that they may require little if any sign language exposure.

## Research Questions

The aim of this study is to examine BSL narratives produced by deaf children with varying degrees of exposure to BSL and adult native signers of BSL in order to identify similarities and differences in the use of predicate types. We were also interested to see if the adult and child signers exhibit different patterns in the use of productive predicate structures (e.g., CA and DCs) in comparison with more conventionalized predicates (e.g., predicates containing only lexical material). In this study we examine narratives of deaf signing children from deaf families, as well as deaf signing children from hearing families and deaf children from hearing families with minimal exposure to BSL, to determine the extent to which early sign language exposure predicts the acquisition of various predicate types in comparison with adult native signers. Given previous sign language and gesture research, we predict that deaf children at age 6 will use all predicate types (including those with minimal BSL exposure) but with less use of simultaneous predicate types compared to deaf adult signers due to a still early developmental stage. We also predict that deaf children from deaf families will use more simultaneous predicate types than deaf children from hearing families due to the latter’s more limited sign language experience.

## Method

### Participants

The study examined the use of predicate types in three groups of severely/profoundly deaf children: (1) five deaf children from deaf families who acquired BSL since birth, (2) five deaf children from hearing families who acquired BSL after age 5 (in bilingual schools where BSL was used as the language of instruction to teach written English), and (3) five deaf children from hearing families with minimal exposure to sign language (in mainstream schools). The aim was to recruit children at around 6–7 years of age, as this was the one of the earliest ages reported in the literature when deaf children begin to master use of entity DCs and/or lexical signs together with CA (e.g., Reilly, 2000; Slobin et al., 2003).^5^ The children were aged between 5;1 and 7;5 (mean age 6;9; median age 6;11), as shown in Table 1. Table 2 shows the alias name for each child participant, their ages at the time of filming in years;months, and their preferred language as reported in a parental questionnaire. For the children with minimal BSL exposure, Table 2 also shows the school’s primary communication method (TC for Total Communication where English is used with some BSL signs at the same time, and O for oral where focus is on speech and speechreading). The adult group consisted of deaf adults who were from deaf families and acquired BSL from birth, as shown in Table 3.

**Table 1:** Child participant summary

	Language background	Mean age	Median age	*N*
DD-C	Deaf children from deaf families who have acquired BSL natively since birth (BSL as preferred language)	6;7	7;0	5
DH-bi	Deaf children from hearing families who have acquired BSL from age 5 in bilingual schools where both BSL and English is used (BSL as preferred language)	6;8	6;5	5
DH-TC/oral	Deaf children from hearing families with minimal exposure to BSL in schools using either oral methods or total communication (English as preferred language)	6;10	6;11	5

**Table 2:** Individual child participant details

DD-C			DH-bi			DH-TC/oral		
Alias	Age	Pref. lang.	Alias	Age	Pref. lang.	Alias	Age	Pref. lang.
Rachel	7;5	BSL	Hunter	7;4	BSL	Ben (TC)	7;3	Not known
Tom	7;0	BSL	Connor	7;0	BSL	Maya (TC)	7;0	English
Gretchen	7;0	BSL	Kyle	6;5	BSL	Millie (TC)	6;11	English
Penny	6;4	BSL	Isabel	6;5	BSL	Andre (O)	6;8	English
Oliver	5;1	BSL	Sally	6;3	BSL	Kendra (O)	6;7	English

*Note.* TC, total communication; O, oral communication.

**Table 3:** Adult participant summary

	Language background	AoA	Age range	*N*
DD-A	Deaf adults from deaf families who have acquired BSL natively since birth (BSL as preferred language)	Birth	22–56	5

### Task and Stimulus Material

The stimuli consisted of short cartoon clips, which were intended to elicit short narratives containing entity DCs and CA. The film clips consisted of an excerpt from the Pink Panther cartoon *Keep Our Forests Pink* (45 s long) and also three short excerpts from the Wallace and Gromit film *The Wrong Trousers*, 25 s long on average.

Participants were asked to watch each of the cartoon clips and afterwards to describe each to a deaf native signer of BSL (S.S., the second author). There were three practice clips shown to ensure that participants understood the instructions.^6^ All signed productions were filmed and analyzed using ELAN multimedia annotation software. In order to elicit narratives that were as natural as possible, no instructions were given about which language or modality should be used. Participants whose preferred language was BSL interacted exclusively with S.S. For participants whose preferred language was English (i.e., the DH-TC/oral child group), sessions were led by S.S. with a BSL/English interpreter present for clarification if needed.

### Coding

**Figure 2:**
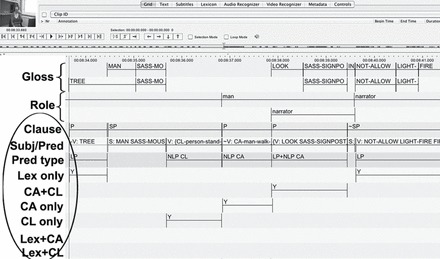
ELAN screen shot showing tiers that were coded.

For the quantitative analysis, signed narratives were first coded using the software package ELAN (http://www.lat-mpi.eu/tools/elan) for CA (or any form of enactment), including which body part(s) were enacting the referent’s body parts, whole entity DCs—that is, any construction where the hand represented a whole entity in order to describe location and/or motion of that referent—and SASS where the signer’s hands trace or outline the size or shape of an entity. Narratives were also coded for lexical elements and conventional gestures. All data were coded by deaf, fluent signers of BSL who were native signers of BSL or another sign language. Secondly, clauses containing minimally a predicative element were identified following the criteria used by Johnston et al. (2007). Predicate construction types were then coded. To ensure consistency and adherence to the coding guidelines outlined below, fluent BSL signers and a deaf native BSL signer checked the annotations. Every narrative described here was coded and/or checked in full by a minimum of three coders; in every case, at least one of the coders was blind to the background characteristics of each child and to the hypotheses of the current study. Disagreements were mutually resolved and agreed by all authors. The tiers are exemplified in Figure 2.

To identify predicates and their arguments, we followed Johnston et al.’s (2007) criteria for determining clausehood. Following their criteria, a clause had to minimally include a predicate acting as a nucleus of the clause. This predicate could have a subject or not (variable subject presence or null subjects/pro-drop is well documented for many sign languages; see, e.g., McKee, Schembri, McKee, & Johnston, 2011; Wulf, Dudis, Bayley, & Lucas, 2002). We then determined whether each predicate included lexical predicates (LPs), non-lexical predicates (NLPs), or both. The following sections provide a description of the predicate types identified in our data.

### NLPs.

#### CA predicate only

**Photo 4:**
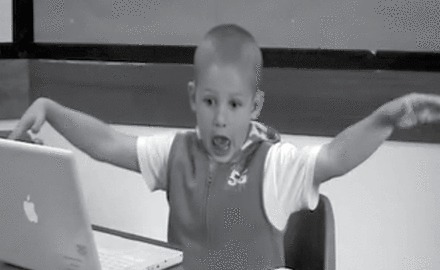
CA predicate where the signer’s body (head, face, arms, and torso) represent Wallace balancing on a train.

We created a CA-only tier in order to identify all manual and non-manual stretches of CA or enactment without any use of DC or lexical items within the same predicate. This tier served to identify predicates in which the participant used the face, head, hands, and/or body to represent corresponding parts or actions of the referent in real-world space, without the use of highly conventional (i.e., lexical or action gesture) elements within the same predicate.^7^ Photo 4 shows an example of enactment of the head, face, torso, and arms. These were not the only articulators that could be/were involved in the children’s use of CA. Some children used their entire bodies for enactment (e.g., lying down to represent the character lying down); for the purposes of this study, which aims to examine enactment in its various forms without bias about what may be considered “linguistic” or “gestural,” these were also coded as “CA only.”

Arm/hand tiers, one for the dominant and one for the non-dominant arm/hand, were coded when the arm and/or hand of the participant was used to represent the arm and/or hand of the referent. This included any productive uses of handling or manipulative constructions that represented the hand(s) of the referent handling or manipulating an object and also any movements of the arm(s).

#### DC only

**Photo 5:**
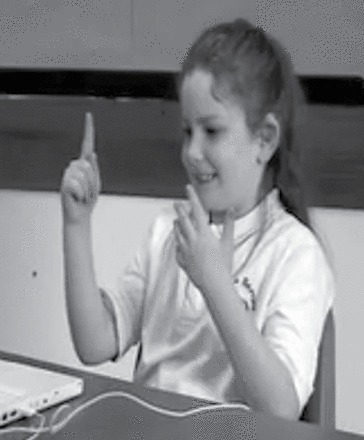
Whole entity construction. The signer’s right hand represents the Pink Panther arriving.

The “DC only” tier was used to identify whole or part entity DCs when one or both hands were used to represent the location and/or motion of all or part of an entity (e.g., animate entities such as person, animal, car, or inanimate entities such as a wall, motorbike), or SASS, which traced the outline of an object, such as a signpost, without any simultaneous use of CA or lexical items (or action gesture elements). Photo 5 shows an example of a whole entity DC. Although many sign language researchers consider handling constructions as types of DCs, we included handling constructions as types of CA, because in this dataset there was no evidence that the handling handshapes produced were phonemic/categorical. (Also, every token of handling in our data co-occurred with one or more non-manual elements of CA.)

#### CA and DCs

**Photo 6:**
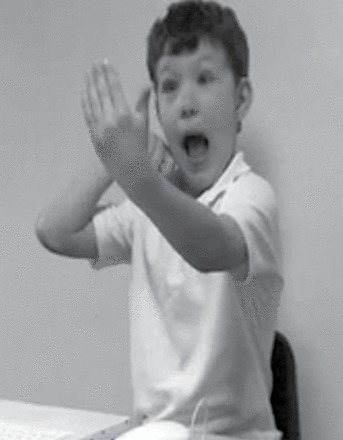
Whole entity constructions with simultaneous CA. The signer’s face represents Wallace (CA) and his hands represent Wallace and the wall (DC).

In addition to the use of predicates with only CA or only DC, we also found predicates in which the signer used DC with CA within the same predicate, either sequentially or simultaneously, to describe the location, motion, or action of a referent, without any lexical or action gesture elements in the same predicate. Photo 6 shows an example of CA used with a whole entity DC. Other examples included simultaneous use of CA on the signer’s face to represent the dog while using the two hands to represent the dog’s ears (part entity constructions).

### LPs.

**Photo 7:**
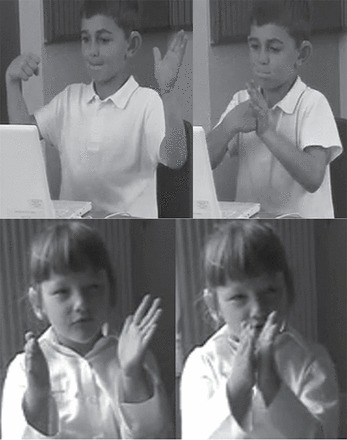
Two representations of the action of Wallace hitting the wall.

Tokens of a predicate without DC and/or CA where the signer used lexical signs or conventionalized gestures to express verbal meaning but not movement or locations of referents in space nor action in real space were coded in the Gloss tier. Those functioning as predicates were also coded in the Sign only (Lex Sign) tier. These included lexical BSL verbs (plain, agreement, or indicating verbs—e.g., the BSL plain verb PLAY) or predicative nominals and adjectives. Conventional verbal gestures were included in this category where the location and/or movement of the gesture did not appear to productively reflect the location and movement of the referent. For example, the examples shown in Photo 7 were both used to describe the action of Wallace hitting the wall. Unlike the example shown in Photo 6 where the dominant hand representing Wallace moved toward the stationary non-dominant hand representing the wall (where movement and location were highly iconic), the children’s two hands in both tokens shown in Photo 7 come together to meet. This suggests that neither hand represents the wall as directly as in Photo 6. Furthermore, because some productions that could be considered lexical signs in BSL also could be used as gestures by non-signers, such as those shown in Photo 7, we made no attempt to distinguish lexical signs from gestures with verbal meaning within the LP category. In cases where the production could be ambiguous between a LP and a DC (e.g., productions ambiguous between the BSL sign WALK and the related whole entity construction from which it has been lexicalized, depicting a two-legged entity moving along a surface), we used criteria outlined in Cormier et al. (2012).

### Predicates containing both lexical and non-lexical elements.

A type of a complex predicate containing lexical and non-lexical material articulated simultaneously or sequentially with verbal meaning was also observed in our data. This category of predicates was further subdivided into three different types.

#### Lexical sign + CA + DC

This predicate type contains tokens of lexical signs (or action gestures) with DCs and CA articulated sequentially or simultaneously. In one example of a combined lexical and non-lexical predicate, the signer describes a motorcycle going over the cliff, with the BSL lexical sign BAD interrupting the start and end of the DC for motorcycle and accompanied by CA depicting the character Gromit.

#### Lexical sign + CA

**Photo 8:**
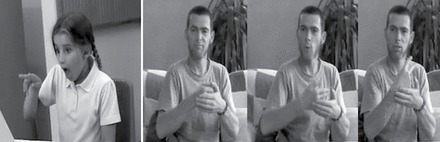
(a) Predicate containing lexical sign LOOK-AT and simultaneous constructed action. (b) Predicate containing lexical sign WALL followed by DC depicting Wallace hitting the wall.

This predicate type commonly contained lexical signs with CA that could be articulated simultaneously or sequentially. Photo 8a shows the BSL verb LOOK-AT with CA where the signer’s face is enacting the Pink Panther.

#### Lexical sign + DC

This predicate type contains lexical material articulated alongside (or simultaneously) with DC. Photo 8b shows an example of a predicate describing Wallace hitting the wall where the lexical sign WALL and a DC representing Wallace are combined.

## Results

### Quantitative Analysis: Frequency of Predicate Types Across Groups

**Figure 3:**
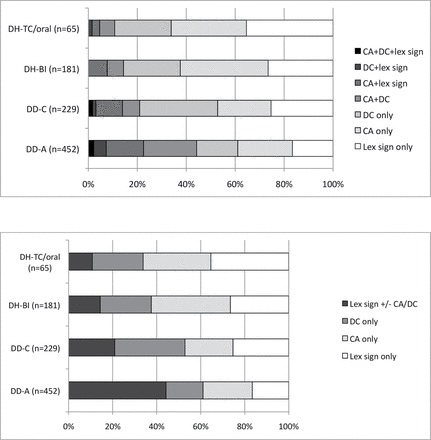
(a, top) Proportions of predicate types used in the narratives across four groups of participants, with simultaneous construction types separated. (b, bottom) Proportions of predicate types used in the narratives across four groups of participants, with simultaneous construction types combined.

The first analysis examines the frequencies of types of predicates used in three groups of deaf individuals—deaf native signing adults (DD-A), deaf native signing children (DD-C), and deaf signing children from hearing families in bilingual schools (DH-bi)—in order to determine the relationship between the amount of exposure to BSL and predicate type. Proportions for all groups (including deaf children with minimal BSL exposure in oral/total communication schools, DH-TC/oral) are shown in Figure 3(a). Due to low cell count, predicates containing both lexical and non-lexical material, for example, lexical signs with DCs and/or CA (i.e., predicates containing both lexical and non-lexical elements) were collapsed into a single category “LexSign +/− CA/DC” in order to carry out the analysis described below, as shown in Figure 3(b).

The deaf children from hearing families with minimal exposure to BSL (i.e., the DH-TC/oral group) were excluded from the quantitative analysis below. Two of the DH-TC/oral children produced spoken language simultaneously with gestures. This was not comparable with the signing/gesturing produced by other children in the same group or in other groups, because speech (including LPs) naturally co-occurs simultaneously with various types of visible gesture (McNeill, 1992), but gesture used without speech in non-signers is not typical (Goldin-Meadow, McNeill, & Singleton, 1996). The remaining three non-speaking DH-TC/oral children were excluded from the analysis due to low cell count for some predicate types (cf. Figure 3a and b). We provide a qualitative analysis of data from all the child participants (including the speaking DH-TC/oral children) in the following section.

**Figure 4.**
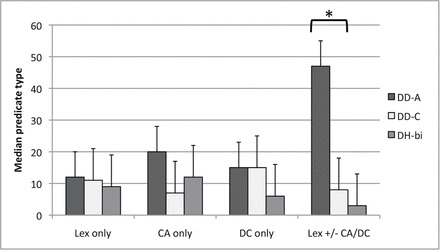
Medians of predicate types used in the narratives across three groups of participants, with simultaneous construction types combined.

A Kruskal–Wallis test was conducted to evaluate differences across the three remaining groups in their use of various predicate constructions (LP only, CA only, DC only, and Lex sign +/– CA/DC). The results indicate that there was a significant difference across the groups in the use of the combined predicate (Lex sign +/– CA/DC), *H* (2, *N* = 15) = 9.26, *p* < .05, *r* = .44, as shown in Figure 4. More specifically, adult signers used predicate structures in which lexical signs were accompanied by CA and/or DC or both (i.e., the category “Lex sign +/− CA/DC” in Figure 3b, broken down into categories “CA + DC,” “CA + lex sign,” “DC + lex sign,” and “CA + DC + lex sign” in Figure 3a). Of all the combined predicate types, the largest proportions in any given group were CA + DC and CA + lex sign. The differences across groups in the use of other predicates were not significant; the groups did not differ in the use of LP only, *H* (2, *N* = 15) = 1.004, *p* > .05; CA only, *H* (2, *N* = 15) = 3.53, *p* < .05; or DC only, *H* (2, *N* = 15) = 3.78, *p* < .05. The proportion of variability in the ranked dependent variable accounted for by the amount of exposure to BSL variable was .44, indicating a strong relationship between the amount of BSL exposure and use of combined predicates.

Further, pairwise comparisons using the Mann–Whitney *U* test were conducted to evaluate differences across the three groups, controlling for Type I error across tests by using the Bonferroni approach. The test indicated that deaf adults used significantly more combined predicates than DD-C, *U* (1, *N* = 5) = 1.00, *Z* = −2.40, *p* < .05, and than DH-bi, *U* (1, *N* = 5) = .00, *Z* = −2.61, *p* < .05. The two child groups did not differ from each other, *U* (1, *N* = 5) = 7.5*, Z* = −1.05, *p* > .05. Table 4 provides a summary of the medians and mean ranks of predicate types for each group.

**Table 4:** Medians (and mean ranks) of predicate types in each group

	DD-A	DD-C	DH-bi
Lex only	12 (9.5)	11 (7.8)	9 (6.7)
CA only	20 (10.9)	7 (5.7)	12 (7.4)
DC only	15 (9.8)	15 (9.3)	6 (4.9)
Lex +/− CA/DC	47 (12.8)	8 (6.7)	3 (4.5)

**Figure 5:**
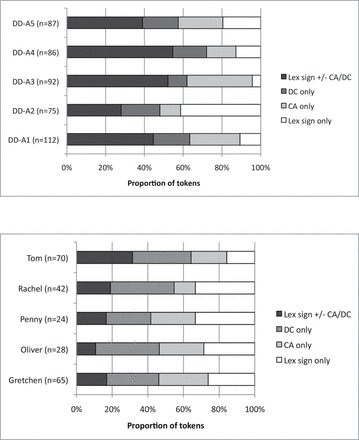

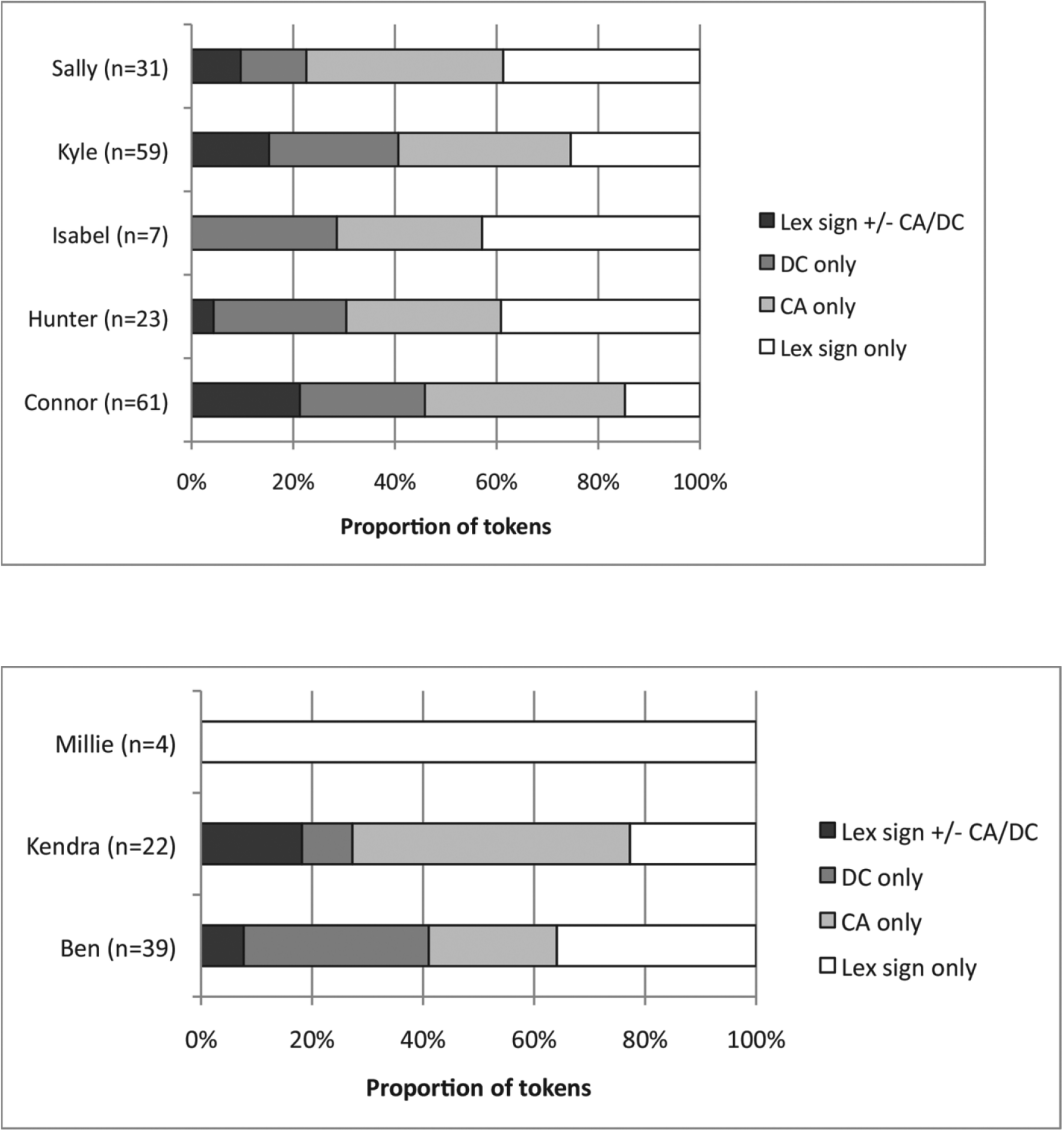
(a, top) Proportions of predicate types used in the narratives of adults (DD-A, by participant), with simultaneous construction types combined. (b, bottom) Proportions of predicate types used in the narratives of deaf children from deaf families (DD-C, by participant), with simultaneous construction types combined.Figure 5 Continued. (c, top) Proportions of predicate types used in the narratives of deaf children from hearing families in a bilingual school (DH-bi, by participant), with simultaneous construction types combined. (d, bottom) Proportions of predicate types used in the narratives of deaf children from hearing families with minimal BSL exposure (DH-TC/oral, by participant), with simultaneous construction types combined.

Production of predicate tokens of each participant is plotted (by group) in Figure 5a–d. Examination of these individual patterns reveals considerable variation in the patterns across children, even within groups. Even so, all of the native signing children produced some simultaneous constructions, whereas the same cannot be said of the DH-bi and DH-TC/oral groups. Furthermore, the children who overall produced the least number of predicate tokens relative to the other children (i.e., DH-bi Isabel and DH-TC Millie) also did not produce any combined predicates.

### Qualitative Assessment of Child Data

In addition to the quantitative analysis, here we provide some descriptive qualitative analysis to give some impressions of the narratives produced by the groups (and by individual children).

#### DD-C.

Overall the deaf children from deaf families in bilingual schools (DD-C) gave more detailed and more complete narratives compared to the other children, particularly Rachel, Tom, and Gretchen. The narratives of these three children appeared the most adult-like in terms of events described, order of events, and identification of referents. Oliver was the youngest participant by far (5;1) and his narratives were less well developed than Rachel, Tom, and Gretchen. Rachel, Tom, Gretchen, and Oliver followed the instructions that were given to them at the start—that is, they watched the film clip and then described it to the researcher afterwards. Penny’s narratives were very short and at times she seemed to be signing while still watching the clip.

#### DH-bi.

Of the deaf children from a hearing family in a bilingual school (DH-bi), Kyle was the only one who followed the instructions in describing both clips after watching them. Kyle also gave the most detailed, coherent narratives of the DH-bi group, although his included some embellishments that the other children did not (e.g., within his Pink Panther narrative he enacted the man while in the tent looking for something, though the clip never shows what the man is doing in the tent). Connor’s descriptions were fairly detailed and coherent, though he told some events in the wrong order (his narrative describing the Wallace and Gromit clip began with Wallace hitting the wall, when this is what happened at the end of the clip). Also he seemed to be signing some parts of his Pink Panther narrative while still watching the stimulus clip. It was very clear that Sally and Hunter told both of their narratives while they were still watching the stimulus clips, so they mentioned characters and events, and reacted to them, as they saw them. Isabel watched the clips before describing them, but her descriptions were extremely short and tended to be just a simple summary with one noun sign and a single predicate (e.g., the sign MAN followed by CA showing the man chopping for the Pink Panther cartoon). Like the DD-C group, individual tokens within each predicate type generally appeared adult-like in their articulation and context of use.

#### DH-TC/oral.

The deaf children from hearing families in schools using a total communication or oral communication method (DH-TC/oral) were the most varied group. Andre (from an oral school) gave his narratives orally in English with no noticeable use of BSL lexical signs at all. Maya (from a total communication school) gave her narratives orally in English with some supporting use of BSL lexical signs. Both Andre and Maya used a fair amount of CA/enactment (both iconic gestures co-occurring with their speech and pantomimic gestures produced without any co-occurring speech). Kendra (from an oral school) used a few BSL lexical signs in her descriptions but otherwise used enactment and iconic gestures co-occurring with speech or mouthing. Ben (from a total communication school) used some BSL lexical signs in his descriptions but he signed his narratives while watching the clips. Ben and Kendra both used DCs for representing the location and motion of the vehicles in the Wallace and Gromit clips. Millie (from a total communication school) like Isabel from the DH-bi group, tended to give very short summaries of the stories (e.g., HIT WALL HIT WALL MAN for the Wallace and Gromit clip) and some factually inaccurate/nonsensical descriptions (e.g., in her Pink Panther narrative she produced DOG HIT, then a pause, and then FIRE). Ben’s, Kendra’s, and Millie’s articulation (particularly handshape) was not as clear as the other DD and DH-bi children.

##### DH-TC/oral children who did not use speech

The total number of predicate tokens is shown in Figure 3a and b for this group as 65. Note that this only includes the three children (including Ben and Millie in a school that used total communication and Kendra in an oral school where little/no signing was used) who did not use speech in their narratives, which is one reason why this number is so much lower than the other groups. However, in comparing the proportions of the DH-TC/oral children with the other child groups, there do not seem to be any major differences between the DH-TC/oral group and the other child groups.

##### DH-TC/oral children who used speech

Two of the children—Maya from a total communication school and Andre from an oral school—gave their narratives primarily in spoken English, with some support from gestures and/or a few BSL signs such as MAN. Their productions included the same kinds of predicates as the other groups. Specifically, using the criteria given in the Coding section, we were able to identify their uses of CA only (where the child’s body represented the body of the referent with no simultaneous use of DC or lexical items, as in Photo 4), DC only (where the child’s hand represented the referent with no simultaneous use of CA as in Photo 5), and CA + DC (where the child’s hand represented the referent simultaneously with some non-manual CA as in Photo 6).

However, Maya and Andre’s productions were not included in the analysis shown in Figure 3b because the nature of their LPs, and any CA simultaneously produced with LPs, was quite different from those produced by the other participants not using speech, since their LPs were produced mainly in spoken English. The way that conventionalized and non-conventionalized communication combines within one modality (i.e., the visual-corporal modality) is very different from the way that conventionalized and non-conventionalized communication combines within two different modalities (i.e., the auditory-vocal and visual-corporal modalities), as noted above.

#### Comparison across groups.

There were some tokens of full body CA in DH-TC/oral children—for example, Andre actually stood on one leg and flailed his arms to enact Wallace standing on one leg on the train with his arms flailing.^8^ Otherwise, the use of CA alone looked qualitatively similar across all groups: children and adults. The other predicate types used by DD-C and DH-bi generally appeared adult-like in their articulation and context of use. As noted above, the other predicate types used by the DH-TC/oral children were less clearly formed in their articulation (particularly in handshape), compared to the adults and other child groups. Also, the upright 1-handshape (as in Photo 2) was used by the adults and one of the DD-C children (Rachel shown in Photo 5) to represent the characters arriving on and departing from the scene in the Pink Panther clip, but most of the children used other handshapes (e.g., a lax, unspecified handshape or V-handshape with fingers wiggling to represent a person walking) or other strategies (e.g., CA) to represent these events. This was in contrast to the flat handshape and Y-handshape that was used by most of the children in all groups, as well as the adults, to depict the motion of vehicles (train, motorcycle, sidecar, and lorry) and airplanes, respectively, in the Wallace and Gromit clips.

## Discussion

The main finding of this study was that adult signers used simultaneous constructions in the predicate structures more frequently than the child signers in their narrative descriptions of actions. More specifically, comparison of frequency of predicate types revealed that adult signers (DD-A) differed from the native and bilingual child groups (DD-C and DH-bi) in the use of predicates that combined lexical and non-lexical elements: Adults used proportionately more CA accompanied with lexical items and/or DC than child signers in their cartoon descriptions.

Deaf native signer adults used non-lexical forms such as CA and DC simultaneously or sequentially with lexical elements to predicate referents’ actions in narratives, whereas the children in this study seemed to prefer non-combinatorial constructions, for example, CA alone, DC alone, or lexical signs/action gestures alone. These findings provide empirical support for previous claims that the use of CA simultaneously with lexical signs and/or DC is more complex than other predicate structures (e.g., DC alone or CA alone) and may take deaf children more time to master. This empirical support is based primarily on the frequency of these structures in the children compared to the adults in these narratives. The articulation and context of use of these predicate structures were similar between the DD-C and DH-bi children and the adults. With the DH-TC/oral children, aside from a few tokens of full body CA, their CA alone structures were similar to the adults and other child groups.

The use of CA alone across all groups, including deaf children within minimal BSL exposure, is consistent with previous research pointing to a strong role of enactment in development of sign language and gesture (Loew, 1984; McNeill, 1992). In sign languages, embodiment is always present (in various degrees) and interacts with the lexicon and grammar in complex ways (Liddell, 2003; Taub, 2001). It has been shown that children have embodied understanding of movement and location of referents before they have acquired conventional labels for these concepts (Evans, Alibali, & McNeill, 2001; Howell, Jankowicz, & Becker, 2005). Moreover, production of gesture and subsequently the first word by children between the ages of 10 and 23 months emerged from the child’s action with a corresponding meaning (Capirci, Contaldo, Caselli, & Volterra, 2005; Johnston & Slobin, 1979). It appears that children are able to extrapolate action gestures from functional actions to enactments that represent actions by any referent beyond just themselves from a very early age (McNeill, 1992; Werner & Kaplan, 1963).

All groups of children, including those with minimal exposure to BSL, also used DCs—that is, constructions in which the hand represented all or part of a referent. The handshapes used by the DH-TC/oral children were less clearly formed than those of the other participants, but otherwise the productions were similar, particularly for representing vehicles. For DCs representing people and animals, the handshapes were less clear and less consistent both within and across the child groups. Given the findings of Schembri, Jones, and Burnham (2005) who found that deaf adult signers of Auslan used more conventionalized handshapes in their entity constructions than non-signers did in their action gestures, it may be that at age 6 the entity handshapes of deaf signing children are not yet conventionalized to the degree of deaf adults. Further research is needed to compare a wider range of DCs in signing children and children with little or no exposure to a sign language and to investigate this development at older ages.

Adult signers make use of enactment in different ways than children in that they flexibly and more systematically combine enactment (CA) with lexical elements or DCs in order to construct coherent discourse. Our data show that both adult and child signers combine CA with other non-lexical as well as lexical elements. However, the adult signers combined CA with lexical/non-lexical elements more frequently than the child signers to form complex predicates. This suggests that combining CA with other sign language predicates (DC and/or lexical verbs) increases with sign language experience. This supports previous claims about the complexity of structures using simultaneous CA and DCs (Aarons & Morgan, 2003; Vermeerbergen, Leeson, & Crasborn, 2007) but also extends this to other types of simultaneous constructions. The fact that adults used CA with lexical signs and/or conventional gestures within a single predicate much more than the children suggests that this is also a complex skill. Although simultaneous uses of CA and other lexical and non-lexical constructions (including simultaneous uses of DC and lexical signs) were present in most children’s signing (except DH-bi Isabel and DH-TC Millie who also produced few tokens of predicates overall), both of the DD-C and DH-bi child groups differed proportionately in their use of predicate types from adults, as predicted. However, there was no significant difference in the frequency of use of complex predicates between deaf children from deaf families (DD-C) who would use more complex predicates than deaf children from hearing families (DH-bi); so our second prediction was not borne out. This suggests that the use of complex predicates is not yet adult-like by age 6–7, even in native signing deaf children. Additionally, the children who produced only a few tokens of predicates (e.g., Isabel and Millie) also produced no complex predicates at all. It seems that the ability to use CA simultaneously with other elements may be related to more general communicative competence, cognitive ability, and/or narrative skills common to all deaf children at that age.

It is not clear if this contradicts findings from Reilly (2000) and Reilly, McIntire, and Anderson (1994) who found that deaf children began to use CA (i.e., affective facial expressions representing the referent) consistently and in time with manual lexical signs (representing utterances of referents) at age 6. It could be that the acquisition of simultaneous use of CA and lexical signs when representing utterances, thoughts, or feelings (i.e., quotative CA) begins earlier than simultaneous use of CA and lexical signs when representing action (i.e., non-quotative CA).^9^ However, more research directly comparing these two circumstances is needed to confirm this.

Beyond the comparisons of proportions of predicate types, other than a few tokens of full body CA in the DH-TC/oral children, we found that the use of CA alone looked qualitatively similar across the adult and child groups. The other predicate types used by DD-C and DH-bi generally appeared adult-like in their articulation and context of use. The other predicate types used by DH-TC/oral were less clearly formed in their articulation (particularly in handshape) compared to the adults and other child groups. This suggests that regular exposure to BSL (i.e., more BSL exposure than the DH-TC/oral children are receiving in schools promoting total communication or oral methods) is needed to develop more consistent use of formational parameters, particularly handshapes, in DCs and in lexical signs. Also, the upright 1-handshape was used by several of the adults and one of the DD-C children to represent upright animate entities, but not by the other children who used a V-handshape to represent the legs or simply a lax handshape, whereas the flat handshape and Y-handshape were used by participants in all groups in depicting vehicles and airplanes. Although the degree of iconicity could be argued to be similar across the 1-handshape (for upright being), the flat handshape (for vehicle), and the Y-handshape (for airplanes), and previous research has shown that these entity handshapes are all acquired around the same time by native signing children (de Beuzeville, 2006; Kantor, 1980; Supalla, 1982), it may be that the upright 1-handshape to depict upright animate entities requires some exposure to a sign language in order for its consistent use in DCs to occur. This warrants further research.

Overall, findings support previous studies that claim that the mastery of using CA systematically with other verbal structures occurs in later stages of language development (Emmorey & Reilly, 1998; Reilly et al., 1994). We have shown that the ability to systematically combine CA with other predicates (lexical and non-lexical) is not yet fully developed by age 6–7 in deaf children acquiring BSL natively. The children in this study tended to separate predicate types more often than adults. The differential use of different types of predicates between child and adult groups suggests that coordinating multiple articulators to express verbal meanings through the use of lexical and non-lexical (including embodied) predicates is a complex skill that requires a greater cognitive load and is acquired after age 7. We would predict that children would show increased use of complex predicates (with gradually more use of simultaneous lexical and non-lexical elements) over time after age 7; we leave this issue for future research.

## Funding

This work was supported by the Arts and Humanities Research Council of Great Britain [119360 to K.C. and S.S.] and the Economic and Social Research Council of Great Britain [RES-620-28-6001 and RES-620-28-6002], Deafness, Cognition and Language Research Centre (DCAL).

## Conflict of Interest

No conflicts of interest were reported.

## Notes

1. The category “predicate” in Anderson and Reilly’s (2002) study was defined as the sum of three categories from the MacArthur Communicative Development Inventory for American Sign Language (ASL-CDI): action signs, helping verbs, and descriptive signs.

2.
Kantor (1980) claimed that the data from her study suggested that DCs were mastered around the age of 8–9 years. The discrepancy between findings from Kantor and other more recent studies on DCs is unclear, but in general the lack of consensus amongst researchers as to what constitutes an adult-like form makes comparisons of data across studies difficult.

3. It is not clear whether the predicates with CA in the Emmorey and Reilly (1998) study necessarily included lexical ASL signs or not, given that they do not define their notion of “predicate.”

4. The handshape component of DCs, particularly entity constructions, has been considered the most conventionalized of the major formational parameters, more so than movement or location (Schembri et al., 2005), which is why this part of the lexicon is considered partly lexicalized.

5. This study is part of a larger project on Perspective, Location and Motion in British Sign Language (PaLM). Narrative data have been collected from these same children at ages 8–9 and 9–10.

6. The instructions given to each child were to watch the film clip in its entirety and then to describe the clip to a deaf native signer (S.S., the second author). They were given a chance to practice by watching a practice clip and then signing the description to S.S., and data collection did not commence until it was clear the child understood these instructions. Despite this, quite a few of the children seemed to sign all or part of their narratives while still watching the clips. Obviously this impacted the structure, length, detail, and coherence of their narratives to various degrees. For more information, see the qualitative analysis reported in the Results section.

7. All instances of enactment or CA were coded further in the Eyegaze, Face, Mouth, Torso, Head and Arm/Hands sub-tiers depending on which part(s) of the signer’s body were enacting the referent’s body part(s). This was done in order to reliably distinguish instances of CA from other uses of the body, for example, affective facial expressions reflecting the signer’s own thoughts, emotions, etc. Distinguishing CA from other functions (e.g., affective facial expressions reflecting the signer’s own thoughts or emotions rather than those of the referent or grammatical facial markers) was achieved by a dependency between identification of articulators used for CA and identification of the role that the signer was taking on (Cormier et al., 2011).

8. We have seen at least one token of a DD child (in a dataset of different narratives with the same children reported here) where the child (Gretchen at age 6) used her full body to describe an atypical motion event—that is, an animated cartoon where a woman was gliding face down along a road as if her body was a car. When describing this vignette to a deaf adult, Gretchen physically copied this action by lying down on the ground. Thus, this type of full-body enactment is not something that can be attributed solely to lack of sign language input. Furthermore, it is important to note that patterns that are more “linguistic” (i.e., more conventionalized, restricted to particular articulators, etc.) co-exist in sign languages even in the adult form with more “gestural” (i.e., less conventionalized) forms (Cormier et al., 2012).

9.
Lillo-Martin and Quadros (2011) describe uses of CA in deaf native signing children as young as 2 years of age, arguing that non-quotative CA is acquired before quotative CA. However, it is not clear whether the tokens that they describe co-occur with lexical elements or not; some of their examples glossed as lexical signs (e.g., PUNCH and HIT) would under our analysis be considered to be manual CA. It is also not clear whether they consider the representation of thoughts and feelings (or assumed thoughts and feelings) to be quotative or non-quotative. If the representation of thoughts and feelings could be considered quotative (this would certainly be supported by, e.g., Clark & Gerrig, 1990), then it appears that most if not all the examples they cite of CA used by 2-year-old children could be considered quotative uses of CA.
